# Gait and Functional Movement Quality Improvements Following an Individualized Exercise Program in Pediatric Hemato-Oncological Patients

**DOI:** 10.3390/children13091127

**Published:** 2026-08-23

**Authors:** Linda Peli, Joel Pollet, Eleonora Finazzi, Elisa Inselvini, Vincenzo Pintabona, Nicola Sedaboni, Chiara Gorio, Richard Fabian Schumacher, Fulvio Porta, Massimiliano Gobbo

**Affiliations:** 1 Lega Italiana Lotta Tumori (LILT), 25128 Brescia, Italy; 2 Department of Clinical and Experimental Sciences, University of Brescia, 25123 Brescia, Italy; jpollet@dongnocchi.it (J.P.); massimiliano.gobbo@unibs.it (M.G.); 3 IRCCS Fondazione Don Carlo Gnocchi, 20162 Milan, Italy; 4 Pediatric Oncohematology and Bone Marrow Transplant Unit, Ospedale Dei Bambini, ASST Spedali Civili of Brescia, 25123 Brescia, Italyfulvio.porta@asst-spedalicivili.it (F.P.)

**Keywords:** gait analysis, pediatric oncology, individualized exercise program, Inertial Measurement Unit, principal component analysis, dynamic balance

## Abstract

**Highlights:**

**What are the main findings?**

- Principal component analysis identified four movement domains: Gait Efficiency, Gait Quality, Dynamic Balance, and Functional Abilities.
- Only Dynamic Balance improved significantly after six months of the individualized exercise program.

**What is the implication of the main finding?**

- The improvement in dynamic balance may support better motor control in pediatric hemato-oncological patients.
- Gait analysis can help identify the most relevant movement domains to guide individualized exercise prescription.

**Abstract:**

**Background/Objectives:** Exercise medicine is gaining significant importance in adult oncology to improve physical activity and quality of life (QoL). However, few studies have investigated the effects of adapted physical activities in the pediatric population. The aim of this secondary analysis is to compare the movement quality of a hematological/oncological pediatric population before and after an individualized exercise program (IEP). **Methods:** Participants who met the inclusion criteria (aged 5–18 years, oncological/hematological diagnosis, consent) underwent an IEP delivered both in the hospital and via telemedicine at home. The subject’s movement quality was assessed before and after six months of the IEP using instrumented functional tests (i.e., 6 min walking test, Timed Up and Go, T25-FW, and turning test). To reduce the number of variables obtained, principal component analysis (PCA) was performed, and the resulting scores were then compared. **Results**: A total of 18 subjects (median age 12 years; eight females) were included in the analysis. Through the PCA, four dimensions were identified: Gait Efficiency, Gait Quality, Dynamic Balance, and Functional Abilities. The pre–post comparison showed significant improvements (*p* = 0.002) in Dynamic Balance and a trend (*p* = 0.05) in Gait Efficiency. **Conclusions:** The presented results indicate the crucial Principal Components (PCs) of movement in children treated for cancer. Moreover, in our setting, under the IEP we observed a significant improvement in Dynamic Balance. The results are consistent with those observed in previous studies. However, the limited number of subjects and the study design preclude definitive conclusions.

## 1. Introduction

Although pediatric cancers are considered rare, they are the second leading cause of death among children ages 5–14 years in Europe after trauma [1].

Most, if not all, cancers cause fatigue and pain, reducing physical activity. This can lead to protective postures and muscular hypotrophy, which are often the first symptoms that trigger a diagnosis [2,3].

Luckily, as medical treatments advance, the survival rate has increased significantly. This has led to a shift in research and clinical perspectives towards quality-of-life issues in pediatric oncology patients, with a particular focus on side effects during and after therapy. The most common physical side effects are both acute (e.g., infection, nausea, vomiting, fatigue, loss of appetite, and diarrhea) and chronic (e.g., cardiotoxicity, osteopenia, obesity, and osteonecrosis) [4].

Some of their side effects are specifically associated with motor impairments, such as long-term neurotoxic effects on the peripheral and central nervous systems, demyelination in motor pathways, steroid-induced obesity, and proximal myopathy associated with high-dose corticosteroids [5,6,7]. Furthermore, oncological surgery and radiation therapy can also contribute to motor impairments [8]. These effects can persist for months or even years after treatment cessation, leading to reduced muscular activation, impaired function and balance, and motor difficulties impacting activities of daily living [8]. Children and adolescents are particularly affected because motor skills and their postural coordination are developed primarily during childhood and are essential to children’s physical, cognitive, and social development [9,10].

Numerous studies have also confirmed that children with higher activity levels exhibit improved bone density, reduced obesity and better cardiometabolic risk factors [11,12]. There is evidence of improved body composition, flexibility, and cardiopulmonary capacity, increased strength and endurance in most muscle groups, and evident reduction in cancer-related fatigue [13]. Several studies have demonstrated the safety, feasibility and efficacy of incorporating individualized exercise programs (IEPs) into the therapeutic pathway for cancer patients in terms of physical and psychological health [13]. Identifying the most effective exercise programs to reduce these consequences and their impact on quality of life is a priority. Thus, guidelines [14] and a consensus [15] on exercise interventions in pediatric oncology have been published and are implemented by the FORTEe consortium in their ongoing study [16].

However, few studies have analyzed the various components of movement using specific instruments, such as Inertial Measurement Units [IMU], optoelectronic systems, and surface electromyography, to identify alterations, propose specific IEPs, and assess their efficacy. In this context, principal component analysis (PCA) is a powerful tool for distilling complex sensor-based metrics into interpretable movement components [17].

Objectively collected instrumental gait data is an important step forward in tailoring IEPs, and our pilot study aims to show how these data can be produced and analyzed. Previous studies have adopted PCA to investigate specific functional tests of interest in rehabilitation with the aim of regrouping large amounts of variables into significant components of clinical interest [18].

The primary aim of our study is to evaluate the effect of a six-month IEP by comparing the baseline (T0) and post-intervention (T1) scores of the identified gait Principal Components (PCs). This approach allows us to verify whether the IEP impacts the most relevant dimensions of movement quality specifically. The secondary aim is to identify the PCs that characterize the movement of the identified population.

## 2. Materials and Methods

### 2.1. Study Design and Ethical Considerations

This study was designed as a non-controlled clinical trial (NCCT) integrating an IEP and pre–post clinical evaluations. Outcome assessors were not blinded. The study protocol was approved by the institutional Ethics Committee (protocol #NP3323, 30 October 2018) and informed consent was obtained from parents or legal guardians, with age-appropriate assent from participating children. The study adhered to the ethical principles outlined in the Declaration of Helsinki. The study was registered in OSF: https://doi.org/10.17605/OSF.IO/Z5FPN.

### 2.2. Participants and Settings

Eligible participants were children and adolescents aged 5 to 18 years with a primary or relapse hematological/oncological diagnosis, followed by the Pediatric Onco-Hematology Unit and the Pediatric Bone Marrow Transplant Center of the “Ospedale dei Bambini, Spedali Civili” Hospital in Brescia (Italy).

Exclusion criteria were patients younger than 5 years or older than 18 years; patients with known pre-existing motor disabilities that precluded participation in the IEP; patients with cardiac conditions with an ejection fraction < 40%; missing consent; and insurmountable communication barriers.

The study was open to all types of tumors, stages, and times since diagnosis and treatment regimens. These were not inclusion or exclusion criteria.

Among all children who met the inclusion criteria, only six slots were available at any given time for logistical and organizational reasons. Thus, not all patients could be enrolled immediately after diagnosis. Some patients had to wait for others to drop out or complete the six-month period.

### 2.3. Individualized Exercise Program

The six-month IEP was individually tailored to each participant’s physical capacity, clinical needs, and psychological condition. The program included:

- An aerobic exercise to optimize endurance while minimizing fatigue;
- Resistance training targeting major muscle groups to counteract treatment-related atrophy;
- Balance and coordination exercises aimed at improving neuromuscular control and proprioception.

Before each session, the exercise physiologist and the oncologist verified the absence of contraindications, such as risk of bleeding, anemia, fever, pain, infection, respiratory distress, or other limiting conditions. They then tailored the session accordingly.

Participants ideally completed three 50 min sessions per week of a multimodal exercise program that included aerobic training, resistance exercises, balance activities, and gait-specific exercises focused on gait pattern practice and contralateral interlimb coordination.

Specifically, each training session started with dynamic mobility exercises targeting the major joints. Following the warmup, the main part of the training session included circuit training with upper and lower limb strengthening, core stability, balance, coordination, flexibility and aerobic exercises. Each session concluded with a cool-down consisting of static stretching exercises for the major muscle groups.

During the training sessions, the exercise physiologist checked oxygen saturation and heart rate at least three times: at the start of the session, after a tough exercise, and at the end or whenever necessary to monitor these parameters.

Furthermore, the sessions could be delivered both in person during the child’s hospitalization or via telemedicine through a dedicated web platform (BTS Telerehab—Garbagnate Milanese, Italy) in case the child was at home.

During the six-month study protocol exercise duration, difficulty and intensity were progressively increased whenever the patients’ health conditions allowed.

A more detailed description of the intervention performed is reported elsewhere [19].

### 2.4. Outcome Assessment

Participants were assessed at two timepoints: baseline (T0), defined as soon as possible after enrolment, and after 6 months of intervention (T1), to evaluate longitudinal changes associated with the exercise program.

The movement quality assessment was performed through instrumented clinical functional tests. A single G-Walk was used as Inertial Measurement Unit (IMU) (BTS Bioengineering, Garbagnate Milanese, Italy) positioned on the lower back (L2-S1) through an elastic belt, with a sampling frequency of 100 Hz; this method was chosen in order to provide as much information as possible, and on the other hand to reduce discomfort for the patients to a minimum [20].

Data obtained from the IMU were analyzed using the G-Studio software (3.5.26.0 (2013) version, BTS bioengineering—Garbagnate Milanese, Italy), and the results were then stored in an electronic database and used for statistical analysis.

The functional tests were selected to investigate the main impaired and disabling domains that may be affected in pediatric oncological patients, specifically: balance, gait independence, gait endurance, and functional abilities.

The functional tests performed were the:

- Timed Up and Go test (TUG) for functional ability and balance;
- Turn Test (TT) for functional ability, balance, and gait independence;
- Timed 25-foot walk (T25-FW) at self-selected speed for gait independence;
- 6-Minute Walking Test (6MWT) for gait endurance.

The spatiotemporal variables extracted from the IMU assessments during the TUG included temporal parameters: total time, sit-to-stand, stand-to-sit, walking, intermediate rotation, final turn duration, sit-to-stand and stand-to-sit acceleration parameters (vertical, antero-posterior and lateral). Temporal parameters are indicational functional mobility, transitional movements and dynamic balance, whereas acceleration parameters provide information on movement quality, lower limb efficiency and postural control [21].

For the TT, the variables extracted were speed, cadence, cycle length and duration of initial walk, direction change and final walk. These parameters reflect gait performance, turning ability and motor coordination [22].

The spatiotemporal parameters extracted during the T25-FW were speed, cadence, right and left stance and swing phases, single and double support phases, right and left cycle length and gait symmetry. These variables provide insight into gait efficiency, stability and balance during walking.

A shorter duration of some of these parameters indicate better lower limb muscle function, better functional mobility and walking performance and also improvements in dynamic balance, coordination, postural control and stability [23].

For the 6MWT, the metrics analyzed were distance, speed, symmetry. These outcomes primarily reflect functional exercise capacity, walking endurance and gait efficiency.

An increase in distance indicates an improvement in functional exercise capacity [24].

### 2.5. Statistical Analysis

The statistical analysis was performed using R software (version 4.4.0). The data obtained from the instrumented functional assessment of the recruited sample were presented as means and standard deviations, with medians and interquartile ranges where appropriate. All available data were preliminarily analyzed to impute missing values using the feature’s median, replacing missing values with that median. All features extracted from the instrumented functional assessment were considered; the data were scaled and centered to conduct PCA on the features measured before the IEP (T0). This kind of analysis allowed the summarization of many features into a smaller number of uncorrelated Principal Components (PCs). The number of PCs to retain was determined by considering at least three selection criteria. In particular, we retained the PCs whose actual data exceeded the simulated data, as indicated by Horn’s parallel analysis. Moreover, PCs met the Kaiser–Guttman criterion, the total explained variance exceeded 60%, and the scree plot followed the ‘elbow rule’. As the number of variables will exceed the sample size, the eigenvalues will be calculated according to the Yata et al. method specific for HDLSS cases [25]. The mean communalities of the analyzed variables will be calculated [26]; for each retained PC, Cronbach’s alpha will be calculated from the loadings that reached at least a threshold of 0.5. Each retained PC will be labeled according to the weights of each variable to provide a good synthesis that could also be clinically meaningful. After identification, the PCs’ scores were calculated from the T0 and T1 assessments, and their distribution was tested using the Shapiro–Wilk test. According to the distribution, the paired Student’s T-test and the Wilcoxon signed-rank test were applied. If the number of PCs exceeded 3, the Holm–Bonferroni correction for *p*-values was applied.

## 3. Results

A total of 35 patients were enrolled in the study. Of those, 17 dropped out: ten due to poor adherence by the child or caregiver over the six-month period and seven due to clinical deterioration.

Thus, the study involved 18 subjects with a median age of 12 years (interquartile range [IQR] 9–14), of whom eight were female and ten were male. Anthropometric data showed the following mean z-scores: height for age, 0; weight for age, 0.32; and BMI, −0.05.

Of the 18 patients who completed the six months of IEP, adherence to the full 66 sessions was 79% for a total of 945 sessions (in person 39.9%; via telemedicine 60.1%).

Table 1 lists the subjects who performed the different functional tests based on the instrumented analysis data.

**Figure 1:**
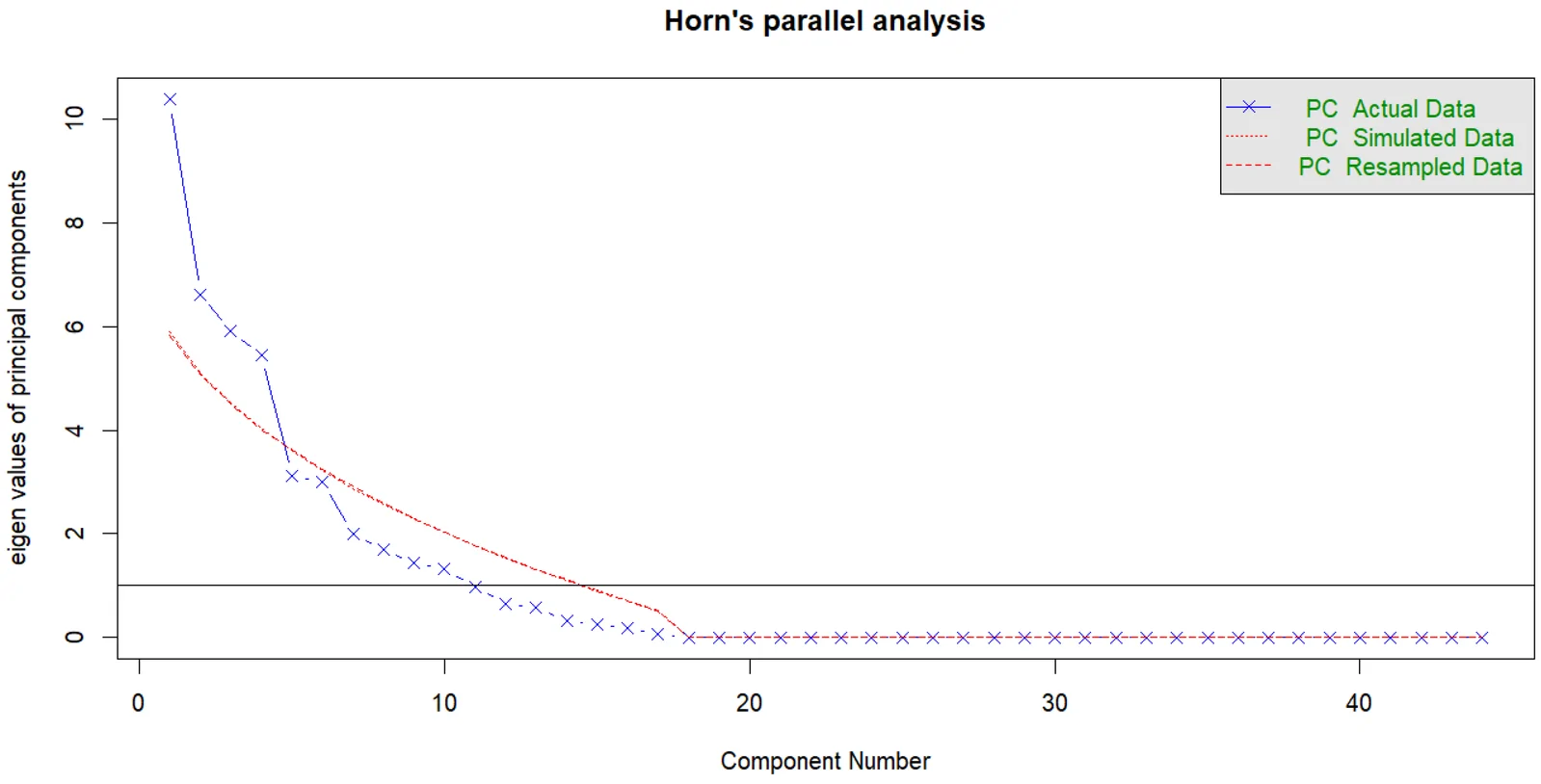
Results of Horn’s parallel analysis.

The PCA was performed following the HDLSS-specific framework of Yata and Aoshima [27]. The resulting eigenvalues exhibited a ‘spiked’ distribution (5207.59, 892.47, and 143.76 for the first three components, respectively), indicating a dominant underlying structure. Although a sharp decline in magnitude was observed after the third component, four components were retained for further analysis in accordance with Horn’s parallel analysis, ensuring a comprehensive capture of the non-random variance in the data. The four retained PCs accounted for 64.5% of the explained variance (PC1 23.6%, PC2 15%, PC3 13.4%, and PC4 12.4%) and were consistent with actual data exceeding those simulated according to Horn’s parallel analysis (Figure 1). The scree plot is presented in Supplementary Figure S1, while in Supplementary Table S1 we reported the Cronbach’s alpha values, eigenvalues, variance, and cumulative variance of each of the retained PCs. The mean communalities of the 44 considered variables were 0.64, and the loadings of each variable for each PC are reported in Supplementary Table S2.

**Figure 2:**
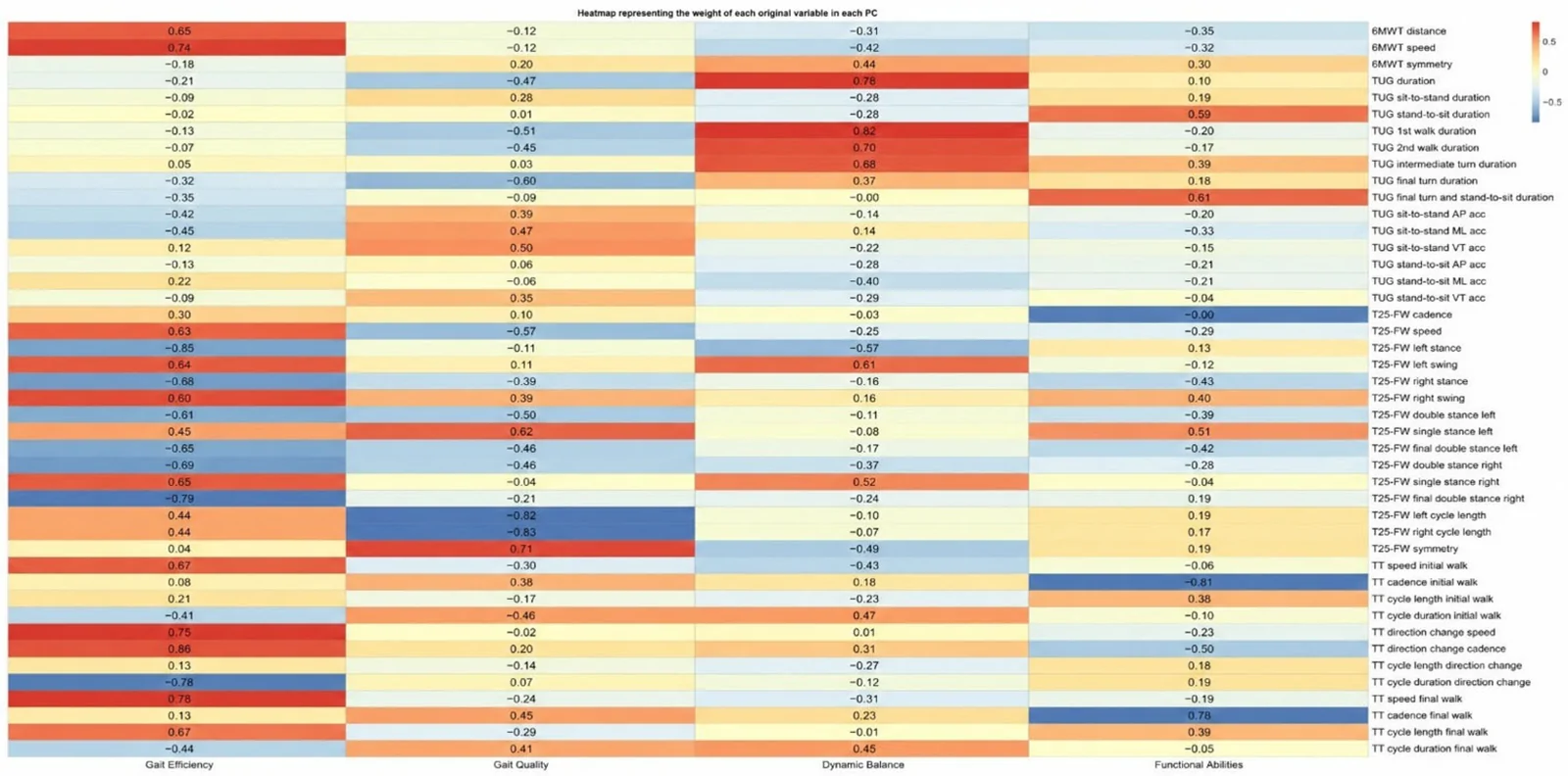
Heatmap representing the weights of each considered variable within each retained PC. Legend: 6MWT: 6-Minute Walking Test; TUG: Timed Up and Go test; T25-FW: Timed 25-foot walk; TT: Turn Test; AP: Antero-posterior; ML: Medio-lateral; VT: Vertical.

The analysis of each feature weight across the four PCs (Figure 2) led us to rename the dimensions as follows: PC1: Gait Efficiency; PC2: Gait Quality; PC3: Dynamic Balance; PC4: Functional Abilities.

The scores for each PC at the two timepoints were calculated, tested for distribution (Supplementary Table S3), and compared. Among the different PCs, only PC3, Dynamic Balance, showed a significant improvement (*p* = 0.002), while the improvement observed in PC1, Gait Efficiency, lost significance after applying the Holm–Bonferroni correction (*p* = 0.054); the other PCs (i.e., PC2 and PC4) were not significant (*p* = 0.834). All the scores and results are presented in Table 2.

Data are presented as mean ± SD or median [1st and 3rd quartiles] according to their distribution, original *p*-values, and adjusted *p*-values. The weight of each variable in every PC is represented in Figure 2.

## 4. Discussion

This secondary analysis of data on the implementation of an IEP for children in treatment for hematological/oncological diseases focused on movement quality aspects. A large number of gait and functional tests were performed using objective instrumental data. To reduce the number of variables (i.e., 44 variables retrieved from the four functional tests performed) and regroup them into meaningful categories to improve the information given to the clinicians while testing those patients, a PCA was carried out. Four PCs were retained based on the eigenvalues, and on the results of the other tests performed (e.g., Horn’s parallel analysis, amount of explained variance, and the analysis of the scree plot). The literature suggests that if the mean communalities exceed 0.6 (i.e., a mean value of 0.64), then retaining four components is appropriate [26].

The comparison of pre- and post-scores from the PCA showed a significant improvement in the ‘Dynamic Balance’ component and a trend in ‘Gait Efficiency’, while the other PCs showed no significant improvement.

The different PCs represent specific gait or functional parameters. The first one represents ‘Gait Efficiency.’ This feature is crucial in everyday life and is primarily represented by gait speed across the various functional tests performed. This PC is fundamental, as many studies have already shown that gait speed is a significant functional marker with clinical relevance [6,28].

The second PC, ‘Gait Quality’, represents the subject’s ability to walk symmetrically with a consistent step pattern and reduced variability. Our findings suggest that although specific gait parameters exhibit individual variability, the overall gait quality is a complex dimension influenced by both treatment-related recovery and musculoskeletal adaptation. Improving gait quality is essential to preventing long-term orthopedic complications and promoting a more natural movement pattern in pediatric survivors [29].

The third PC ‘Dynamic Balance’ encompasses the ability to maintain stability during functional transitions and locomotion. The significant improvement observed in this PC (*p* = 0.002) may indicate the effectiveness of the balance and coordination exercises included in the IEP. However, natural recovery, maturation or placebo effects could also explain the findings. Our results suggest that targeted neuromuscular training can effectively mitigate these deficits.

The fourth component, ‘Functional Ability’, refers to the integration of strength, coordination, and endurance required for complex daily tasks, such as rising from a chair or navigating obstacles. While this component did not show a statistically significant improvement in this preliminary analysis, our observation suggests that more intensive tailored interventions and prolonged resistance training may be required to elicit substantial changes in overall functional capacity [30].

The observed improvement in Dynamic Balance represents a clinically relevant finding because it may indicate enhanced postural control and functional mobility. However, the potential implications of these changes, including a possible reduction in fall risk and increased confidence in physical activities, were not directly assessed in the present study. The IEP’s ability to target this area specifically underscores the added value of multidimensional exercise protocols in the pediatric oncology setting [31,32].

The importance of an IEP cannot be overstated, given the importance of movement quality in every aspect of life. Using precise movement-quality assessments with IMUs and PCA enables clinicians to identify motor deficits that might be overlooked in traditional clinical evaluations. This data-driven approach allows clinicians to customize exercise prescriptions based on each patient’s unique needs, ensuring that the intervention addresses the most relevant functional impairments [16].

### Limits

This secondary analysis has limitations that require attention in future studies. First, the study was designed as a non-controlled trial, which implies that a consecutive sample of highly heterogeneous subjects is recruited and can be influenced by internal and external factors without the possibility of controlling them. Additionally, the high dropout rate reduced our sample size, preventing us from performing subgroup analyses. To improve compliance, analyses should be performed at shorter intervals, especially since some patients had already completed the intensive part of their oncological treatment without experiencing motor impairment and did not want to or need to continue the program. Furthermore, due to the frailty of our patient cohort, not all scheduled functional assessments could be completed. Consequently, missing data were imputed using the median value of each feature. While this approach is robust against outliers and ensures high replicability, it is a simplification. However, this is a preliminary analysis, and the results should be interpreted with caution [33], considering that natural recovery, physiological maturation, or a return to baseline activity levels could also explain the observed improvements [19].

A notable limitation of this study is the high variables-to-subjects ratio in the PCA. With 44 variables and 18 subjects, the number of features exceeds the sample size (p > np > n), which may lead to rank deficiency in the covariance matrix and potentially affect the stability and generalizability of the extracted components [34]. Eigenvalues were calculated according to the Yata et al. method, which is specifically performed for these HDLSS situations [27]. We calculated and considered the communality values, as shown in a previous study, to be a better predictor of component stability [25]. Moreover, this approach was adopted as an exploratory tool to identify latent patterns within movement quality variables in a specifically frail population, where large-scale recruitment presents ethical and practical challenges. These results should therefore be considered preliminary. Future randomized controlled trials with blinded investigators and large enough cohorts to allow disease- and therapy-specific analyses should elucidate the specific mechanisms by which exercise interventions mitigate treatment-related motor impairments. Incorporating larger cohorts and long-term follow-up in these trials will optimize specific rehabilitation strategies and enhance long-term functional outcomes for pediatric cancer survivors.

## 5. Conclusions

Our preliminary data suggests that an individualized exercise program (IEP) based on objective instrumental data may significantly improve dynamic balance in pediatric hematology/oncology patients. Using principal component analysis (PCA) to distill complex gait and functional test data, four key dimensions of movement quality were identified: Gait Efficiency, Gait Quality, Dynamic Balance, and Functional Abilities. The significant improvement observed in Dynamic Balance (*p* = 0.002) highlights the importance of incorporating targeted balance and coordination exercises into the IEP, to address a prevalent impairment in this vulnerable population. While improvements in Gait Efficiency showed a clear trend, the same could not be shown for Gait Quality and Functional Abilities. Overall, the preliminary findings align with previous research, suggesting the positive impact of exercise on the physical well-being, functional ability, and quality of life of oncological patients. Further studies should confirm the results presented in this preliminary analysis.

## Figures and Tables

**Table 1:** Number of subjects who executed each instrumented functional test at the two timepoints.

6MWT		TUG		T25-FW		TURN Test	
T0	T1	T0	T1	T0	T1	T0	T1
15/18	15/18	17/18	18/18	17/18	18/18	15/18	16/18

**Table 2:** Scores obtained at the two timepoints for each PC; note that T0 data were shifted to 0 during PCA execution. Data are expressed in mean ± SD or median [I Qrt–III Qrt] according to their distribution.

Principal Component	T0 Values	T1 Values	Original *p*-Value	Adjusted *p*-Value
PC1—Gait Efficiency	0.00 ± 3.22	2.50 ± 3.92	0.018	0.054
PC2—Gait Quality	−0.24 [−1.08–1.22]	−0.44 ± 2.24	0.417	0.834
PC3—Dynamic Balance	0.00 ± 2.43	−2.45 ± 2.55	0.001	0.002
PC4—Functional Abilities	0.00 ± 2.34	−0.08 ± 1.76	0.424	0.834

## Data Availability

Data is available by contacting the corresponding author.

## Supplementary Materials

The following supporting information can be downloaded at https://www.mdpi.com/article/10.3390/children13091127/s1, Figure S1: PCA’s scree plot; Table S1: Table reporting data specific to each retained component of the PCA analysis; Table S2: Values of the loadings of each variable for the 4 different PCs; Table S3: Data of the Shapiro-Wilk test for the 4 retained dimensions.

## Abbreviations

The following abbreviations are used in this manuscript:

6MWT	6-Minute Walking Test
AP	Antero-Posterior
IEP	Individual Exercise Program
IMU	Inertial Measurement Unit
ML	Medio-Lateral
PC	Principal Component
PCA	Principal Component Analysis
T25-FW	Timed 25-Foot Walk
TT	Turning Test
TUG	Timed Up and Go Test
VT	Vertical
