# Supplementary material for: Gait and Functional Movement Quality Improvements Following an Individualized Exercise Program in Pediatric Hemato-Oncological Patients
Source: Children (Basel). 2026 Aug 23;13(9):1127. doi: 10.3390/children13091127 (PMC13605195; doi:10.3390/children13091127)
Supplement: Supplementary file 1 [file children-13-01127-s001.zip › children-4461625-supplementary.pdf]

Figure S1. PCA's scree plot

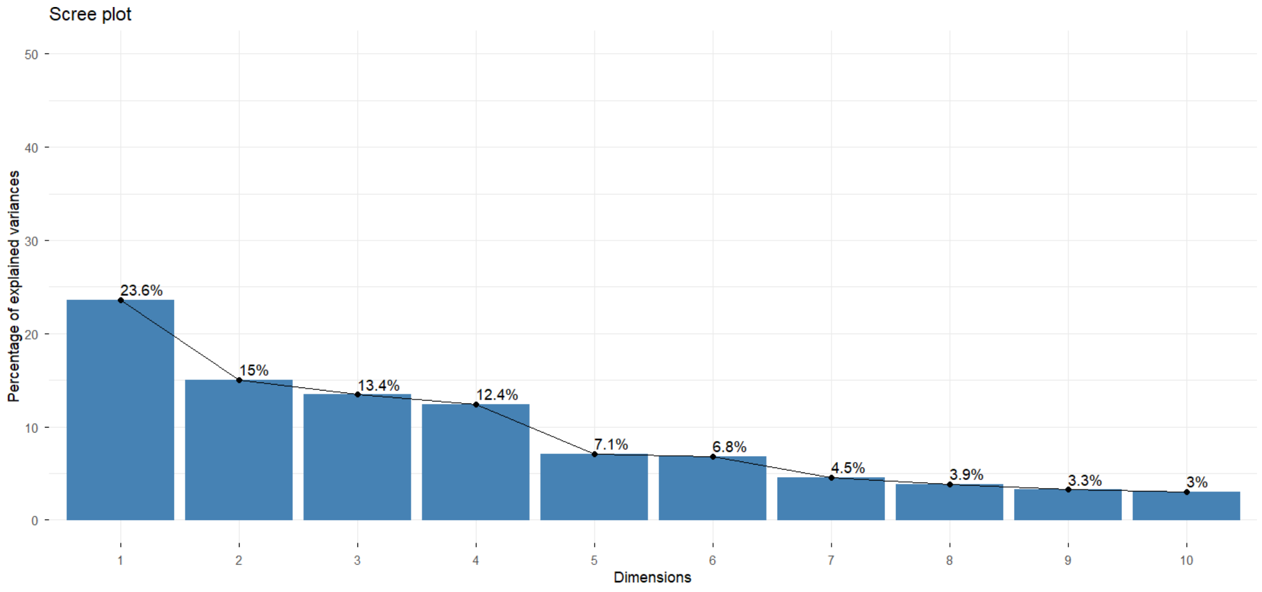

Table S1 – Table reporting data specific to each retained component of the PCA analysis.

Principal Components	Cronbach's alpha	Eigenvalues	Variance	Cumulative variance values
PC1 – Gait Efficiency	0.646	5207.59	23.60	23.60
PC2 – Gait Quality	0.523	892.47	15.05	38.65
PC3 – Dynamic Balance	0.831	143.77	13.44	52.09
PC4 – Functional Abilities	0.762	49.51	12.41	64.50

Table S2. Values of the loadings of each variable for the 4 different PCs.

Variabile	Gait Efficiency	Gait Quality	Dynamic Balance	Functional Abilities
6MWT distance	0.64725795	-0.12340877	-0.313909194	-0.34741638
6MWT speed	0.74097676	-0.12259816	-0.418303570	-0.31834223
6MWT symmetry	-0.17875890	0.20267691	0.439571328	0.29745191
TUG duration	-0.20967148	-0.47245521	0.761574288	0.09543591
TUG sit-to-stand duration	-0.09440257	0.27795326	-0.275871958	0.18805697
TUG stand-to-sit duration	-0.02493221	0.01124449	-0.275550520	0.58837471
TUG 1st walk duration	-0.12906779	-0.50657565	0.783349392	-0.19844988
TUG 2nd walk duration	-0.07212023	-0.44817442	0.695978274	-0.16543639
TUG intermediate turn duration	0.05079449	0.02611323	0.682349598	0.39178269
TUG final turn duration	-0.31716941	-0.59908384	0.370058141	0.17986659
TUG final turn and stand-to-sit duration	-0.35052496	-0.08789913	-0.001259858	0.60698863
TUG sit-to-stand AP acc	-0.42316616	0.38827017	-0.139614113	-0.19826439
TUG sit-to-stand ML acc	-0.45337015	0.46608387	0.137038107	-0.32605639
TUG sit-to-stand VT acc	0.11802201	0.49829330	-0.224616672	-0.14868240
TUG stand-to-sit AP acc	-0.13141510	0.06424340	-0.284330889	-0.20585691
TUG stand-to-sit ML acc	0.21833544	-0.05562258	-0.395535614	-0.21460176
TUG stand-to-sit VT acc	-0.09084293	0.35091798	-0.290699033	-0.04488054
T25-FW cadence	0.30266052	0.09792981	-0.026314168	-0.80081791
T25-FW speed	0.62917042	-0.56801548	-0.246867116	-0.29427095

T25-FW left stance	-0.64967733	-0.11054013	-0.574398166	0.12970001
T25-FW left swing	0.64101298	0.10683687	0.605938993	-0.11855994
T25-FW right stance	-0.68452924	-0.38849623	-0.161436075	-0.42627229
T25-FW right swing	0.69038906	0.38825531	0.155636386	0.40460958
T25-FW double stance left	-0.61259151	-0.49910384	-0.105915255	-0.38735962
T25-FW single stance left	0.44551037	0.62468479	-0.077358217	0.50678696
T25-FW final double stance left	-0.64747448	-0.46002705	-0.165120861	-0.42193559
T25-FW double stance right	-0.69361153	-0.45511662	-0.374954490	-0.27725610
T25-FW single stance right	0.64589824	-0.04015677	0.522301007	-0.03839078
T25-FW final double stance right	-0.79408523	-0.21257098	-0.241508951	-0.18526139
T25-FW left cycle length	0.43754276	-0.81623991	-0.098284286	0.18895260
T25-FW right cycle length	0.43890972	-0.82572121	-0.070762094	0.16794615
T25-FW symmetry	0.04124897	0.71212675	-0.486569291	0.18516974
TT speed initial walk	0.66517479	-0.30332853	-0.427998303	-0.06033445
TT cadence initial walk	0.08384107	0.38458665	0.177946652	-0.80792764
TT cycle length initial walk	0.21340698	-0.17133283	-0.234693658	0.37917933
TT cycle duration initial walk	-0.40872634	0.46169295	0.467047645	-0.09917406
TT direction change speed	0.74522649	-0.01574328	0.005897009	-0.22554556
TT direction change cadence	0.68370044	0.19932072	0.312658926	-0.49567987

TT cycle length direction change	0.12836735	-0.14351729	-0.270619244	0.17698056
TT cycle duration direction change	-0.78267953	0.06517212	-0.122949284	0.18809140
TT speed final walk	0.76411056	-0.23956227	-0.308626053	-0.18945221
TT cadence final walk	0.12803248	0.45166098	0.226620418	-0.78452833
TT cycle length final walk	0.57428845	-0.29300031	-0.010979635	0.39007485
TT cycle duration final walk	-0.44215925	0.40666198	0.452895831	-0.04770569

Table S3- Data of the Shapiro-Wilk test for the 4 retained dimensions.

PC1 – Gait Efficiency				PC2 – Gait Quality				PC3 – Dynamic Balance				PC4 – Functional Abilities			
T0		T1		T0		T1		T0		T1		T0		T1	
W Score	p-value	W Score	p-value	W Score	p-value	W Score	p-value	W Score	p-value	W Score	p-value	W Score	p-value	W Score	p-value
0.96	0.56	0.86	0.01	0.89	0.03	0.98	0.97	0.96	0.70	0.96	0.67	0.96	0.57	0.94	0.28
